# The effect of Sleep Leadership Training on U.S. Army human performance team members

**DOI:** 10.3389/frsle.2024.1351691

**Published:** 2024-04-08

**Authors:** Talia Barrow, Bryan Yu, Carly Cooper, Elaina DaLomba, Brian Gregg, Maria Barefield, Jon Umlauf

**Affiliations:** ^1^U.S. Army-Baylor University Doctor of Science in Occupational Therapy Program, Brooke Army Medical Center, Fort Sam Houston, TX, United States; ^2^U.S. Army-Baylor University Physical Therapy Doctorate Program, U.S. Army Medical Center of Excellence, Joint Base San Antonio, Fort Sam Houston, TX, United States

**Keywords:** sleep, leadership, military, holistic health, sleep procrastination, military sleep assessment

## Abstract

Most U.S. Servicemembers (SMs) get less than the recommended amount of sleep per night, which has been shown to be directly influenced by local leadership. Previous research demonstrated that a brief sleep leadership training (SLT) protocol resulted in improved knowledge and attitudes regarding sleep among U.S. Army leaders, and improvements in the sleep health of their SMs. Human Performance Teams (HPT) offer one solution to propel the cultural change related to sleep, however, little is known about HPT members' own sleep related knowledge, practices, attitudes, and beliefs. This mixed-methods study determined the effect of (SLT) on the sleep-related knowledge, practices, attitudes, beliefs, and perceived competency of HPT members, explored their experiences addressing SM sleep, and gauged their perceived value in receiving the training. Thirty-one individuals received 2 days of SLT. Baseline and 6-week post training follow-up measures were collected, and participants were invited to participate in semi-structured interviews to explore their unique experiences addressing sleep, as well as their perceived value in receiving SLT. Training had no significant effects on HPT members' sleep practices or sleep beliefs, some significant effects on their attitudes and knowledge about sleep, and significant effects on their perceived competence to address sleep in their units. Results suggest that HPT members benefited from the training through enhancement of their competence to address sleep with leadership. However, they struggle to obtain sufficient sleep themselves. More research is needed to identify methods of overcoming specific barriers to sleep imposed on SMs.

## 1 Introduction

The Department of Defense's 2018 Health Related Behaviors Survey indicates that between 54–69% of Servicemembers (SMs) report getting < 6 h of sleep per night during the workweek, with 31% of those getting 5 h or less (Harrison et al., 2022). Insufficient sleep in a population which the nation relies upon to be *combat ready* can significantly hamper the higher cognitive skills that are vital for combat effectiveness (LoPresti et al., 2016; Petrofsky et al., 2022). Additionally, impaired sleep corresponds with suicidal ideation and other behavioral health conditions in the military population including post-traumatic stress disorder, generalized anxiety disorder and major depression (Taylor et al., 2014).

Rates of insufficient sleep are high in SMs both with, and without deployment experience (Troxel et al., 2015). This indicates that factors unique to the military but unrelated to war, may contribute to sleep difficulties, or barriers to obtaining sufficient sleep (Troxel et al., 2015). Another factor unique to SMs is the encroachment of leadership into one's personal life (Gunia et al., 2015). Cultural norms related to sleep in a military unit may be influenced by a leader's own attitudes, beliefs and/or lack of knowledge about sleep. Because of this, subordinate SMs may have little personal control over their own sleep routines.

Studies suggest that good leadership skills can at least partially buffer against the barriers to sleep that SMs encounter (Gunia et al., 2015). Army doctrine states that “while good leadership is essential for a wide range of unit outcomes, leadership behaviors that target sleep can improve the sleep habits of unit members and the unit's overall culture” (FM 7–22, p. 11–5). In fact, a 2021 randomized controlled trial by Adler et al. (2021) found that a brief sleep intervention, SLT, designed to educate mid-level Army leaders about sleep health resulted in their subordinates being more likely to sleep 7 or more hours per night when compared to subordinates assigned to leaders in the control group. The study concluded that leadership focused training interventions may “be able to shift sleep health and the cultural perspective on sleep” (Adler et al., 2021, p. 29). The authors recommended that future research focus on expanding upon ways to target cultural change and to reinforce sleep education to Army leaders over time.

One solution to reinforce education and to contribute to cultural change is to ensure that all Army healthcare providers are competent to address sleep. A study by Abdelwadoud et al. (2022) which examined the perceptions of key military stakeholders including economic-decision makers, primary care managers and SMs themselves found the current state of military sleep management practices to be neither satisfactory nor maximally effective. The authors noted many barriers to managing sleep in the military population including a lack of sleep knowledge among healthcare providers, the need for more qualified providers capable of addressing sleep, the need for behavioral approaches to address sleep, and the need for standardized sleep education materials and tools for provider utilization.

To address some of these issues, needed cultural changes, and other problems related to SM health and wellbeing, the Army recently implemented a system of care known as Holistic Health and Fitness (H2F). H2F represents a paradigm shift from a reactive to a proactive system of care that now formally recognizes non-physical domains of health, including sleep, as vital to wellbeing and, equally as important to health as physical preparedness. H2F programs consist of human performance team (HPT) members from varied fields including physical therapy, dietetics, occupational therapy, certified strength and conditioning, athletic training, and cognitive performance, to provide a more holistic approach to care than what was historically valued by the Army.

These novel, multidisciplinary groups of allied health and performance professionals, embedded throughout the Army, offer one solution to address sleep issues in this population. However, little is currently known about the sleep related knowledge, practices, attitudes, beliefs, and competency of HPT members to address sleep. In fact, much of the literature suggests that healthcare providers receive minimal education related to sleep and are inadequately prepared by their educational programs to address sleep (Ye and Smith, 2015; Meaklim et al., 2020). Further, healthcare providers themselves are considered a population at increased risk for sleep deprivation (Siddalingaiah et al., 2017; Parry et al., 2018). Some evidence suggests a decrement in the performance and productivity of healthcare providers who suffer from sleep deprivation, making them less effective at performing their assigned duties (Shaik et al., 2022). Thus, the present study sought to determine the current state of the sleep-related knowledge, practices, attitudes, beliefs, and perceived competency of HPT members, to determine the effects of SLT on these characteristics and to explore the experiences of HPT members addressing sleep in this innovative system of care. We hypothesized that SLT would affect the sleep characteristics and perceived competence of HPT members.

## 2 Materials and methods

### 2.1 Design

This study utilized a quasi-experimental, concurrent triangulation mixed methods design. Quantitative and qualitative data were collected during one phase and weighted equally. Data were analyzed separately and then compared to assess for convergence, differences, or a combination. Data were then analyzed collectively to determine existing relationships. This study was approved by the San Antonio Institutional Review Board (IRB #957424).

### 2.2 Subjects

HPT members in various H2Fs throughout the continental United States were scheduled to participate in SLT. Participants enrolled in the training were recruited via flyers and e-mail briefings. Inclusion criteria were: (1) current Army HPT members (2) age 17–64 and (3) can read, speak, and understand English. Exclusion criteria were (1) self-reported diagnosis of insomnia, (2) self-reported diagnosis of obstructive sleep apnea, (3) currently taking any prescription sleep medications, (4) unable to complete all aspects of the study and (5) unwilling to disclose information on outcome measures.

### 2.3 Study procedures

**Figure 1:**
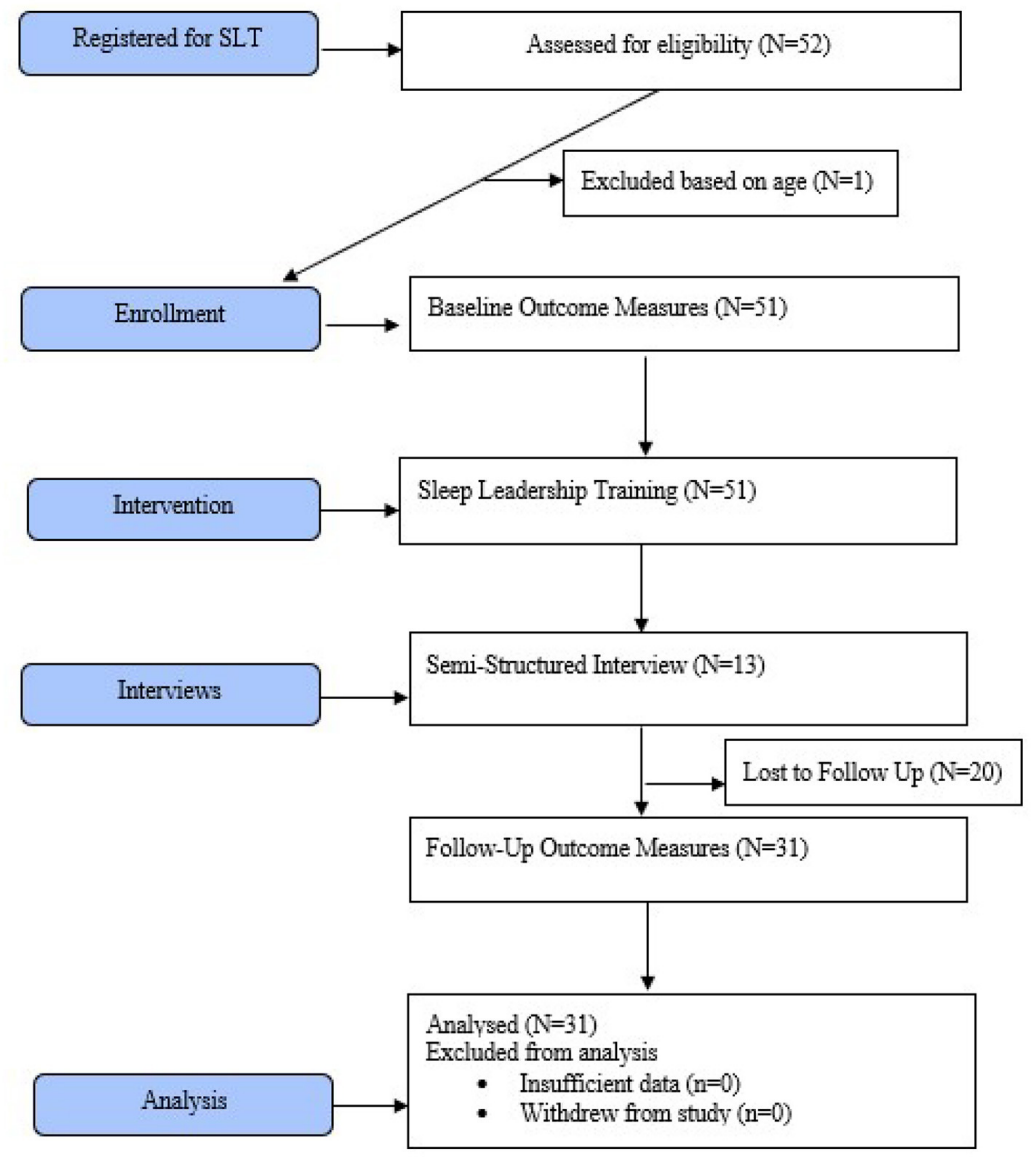
CONSORT diagram demonstrating methodological procedures.

Figure 1 depicts the methodological procedures outlined in this section. Research subjects interested in participating in this study notified the research team and were pre-screened based on eligibility requirements. Eligible subjects were emailed a Qualtrics weblink to complete the questionnaire packet. The packet contained basic demographic data and (1) components of the Sleep Practices and Attitudes Questionnaire (SPAQ), (2) questions related to sleep leadership knowledge and attitudes, and (3) a sleep leadership perceived competence scale. The outcome measures took ~10–15 min to complete, and participants were able to complete them on their home computers or mobile devices.

Following completion of the packet, standardized SLT was provided by instructors who had received specialized SLT training from the Walter Reed Army Institute of Research (WRAIR) (Adler et al., 2021). The training was conducted virtually, using Microsoft Teams, over 2 days. Following the training, participants were invited to participate in telephonic semi-structured interviews, related to their experience addressing sleep in their brigades, and in their experience receiving SLT. Purposive sampling continued until saturation was reached. Each interview was audio-recorded and transcribed verbatim. Six weeks following the completion of the training participants received a follow-up Qualtrics web links consisting of the same measures.

### 2.4 Measures

Sleep knowledge and attitudes were measured using 15 items based on a previous literature assessing the impact of SLT on military leaders (Adler et al., 2021). Participants were asked to indicate their strength of agreement for eight items which measured sleep knowledge and seven items which measured sleep attitudes (1 = strongly disagree to 5 = strongly agree). In keeping with the original research, responses to all 15 questions were categorized such that a score of four or five were rated as agreement with the statement. No psychometric properties have been reported for these questions (Adler et al., 2021).

Sleep practices were assessed using the Sleep Duration, Sleep Debt, Sleep Quality and the Sleepiness/Tiredness Subscales from the Sleep Practices and Attitudes Questionnaire (SPAQ) (Grandner et al., 2014). Sleep duration was assessed by asking HPT members how many hours of sleep they achieve on weekdays/ workdays, and how many hours of sleep they achieve on weekends/ vacation days. Sleep debt was assessed by asking HPT members how many hours of sleep they think they need each night and then subtracting the average number of sleep duration from their identified sleep needs as described in Grandner et al. (2014). A mean sleep quality score was established by asking HPT members to rate their quality of sleep over the last week, as well as their strength of agreement with the statement “I have difficulty related to sleep,” on five-point scales. A mean subscale score for sleepiness/ tiredness was determined by using three dichotomous items (yes/no) to determine if they are sleepy/ tired/ refreshed throughout the day, and one item rating their strength of agreement with the statement, “I never feel sleepy.” Sleep beliefs were assessed using the sleep and health subscale from the SPAQ. This subscale has been used in previous research to determine beliefs about sleep health and asks respondents to rate their degree of belief about how sleep loss affects 15 different variants of their health (Khader et al., 2021). All items were rated on a five-point scale. Most subscales in the SPAQ demonstrate moderate to high internal consistency (α = 0.25 to 0.86), when estimated in a population of 124 participants aged 18–80 years (Grandner et al., 2014). Concurrent and divergent validity were demonstrated by comparing various subscales to existing measures (Grandner et al., 2014).

Perceived competence to address sleep was assessed with four items that were created for this study using a modifiable perceived competence scale (Williams et al., 1998). In previous studies, modified versions of the scale demonstrated excellent internal consistency with Cronbach's alpha between 0.80 and 0.94 (Williams et al., 1998). For the current study, items were designed to capture the perceived competence of HPT members to address sleep with Army leaders and were rated on a 7-point scale (1 = not at all true to 7 = very true). Responses were categorized such that a score of five, six or seven were rated as agreement with each statement.

#### 2.4.1 Data analysis

Descriptive statistics were used to summarize demographic data. All quantitative variables were analyzed in Statistical Package for Social Science (SPSS^®^ version 29.0.0.0) using non-parametric tests. Raw pre-training and post-training data for each question pertaining to sleep practices, knowledge, attitudes, beliefs, and perceived competency related to sleep were examined for any significant differences using Wilcoxon Signed Ranks Tests. These are displayed in Table 2, along with associated significances and percent agreement for some variables.

Qualitative data from the semi-structured interviews were analyzed using semantic thematic analysis following the Braun and Clarke (2006) method, and was managed using NVivo^®^ software (release 1.7.1).

## 3 Results

### 3.1 Demographics

Demographic items were included to identify any potential covariates. Demographic variables consisted of age, gender, profession, employment status, year of graduation, sleep education status and sleep supplement or over the counter medications (Table 1). Seventy-one HPT members who signed up to complete SLT were briefed about the study. Fifty-one HPT members were enrolled in the study, completed baseline measures, and received 2 days of SLT. Twenty HPT members were lost to follow up. A final sample of 31 pre/ post-test results were statistically analyzed. Purposive sampling for qualitative interviews continued until saturation was reached. Thirteen interviews were conducted and qualitatively analyzed.

**Table 1:** Demographics for HPT members.

	**Total participants (*n* = 31)**
**Sex**	
Male	14
Female	17
**Ethnicity**	
White	25
Asian	2
Black or African American	3
Native Hawaiian or Pacific Islander	1
**Status**	
Enlisted	3
Officer	13
DA Civilian	11
Government Contractor	4
**Age**	
22–34	18
35–44	8
45–54	1
55–64	4
**Profession**	
Athletic trainer	1
Certified strength and conditioning specialist	2
Cognitive performance specialist	2
Registered dietitian	1
Dietitian assistant	1
Occupational therapist	15
Occupational therapy assistant	6
Physical therapist	2
Physical therapy assistant	1
**Graduation year**	
< 5 years of practice	11
>5 years of practice	20
**Sleep education**	
Formal education	8 (26%)
No formal education	23(74%)
**Caffeine use**	
Yes	26(84%)
No	5 (16%)
**Non-rx meds/ supplement use**	
Yes	12(39%)
No	19(61%)
**Weeknight sleep at baseline**	
>7 h	18(58%)
< 7 h	13(42%)

Demographic analysis revealed that most participants were in the occupational therapy profession (68%) (the profession responsible for sleep readiness in the military), had been practicing more than 5 years (65%) and had received no formal education related to sleep in their degree granting programs (74%). At the time baseline measurements were collected, 39% of HPT members used a non-prescription sleep aid, or supplement to promote sleep in their own lives and, 42% were getting < 7 h of sleep per night on weeknights.

### 3.2 Sleep knowledge and attitudes

Comparisons in sleep-related knowledge and attitudes are provided in Table 2. Aside from one sleep knowledge question pertaining to the normalcy of Soldiers waking throughout the night (*p* = 0.006), and one sleep attitude question pertaining to a Soldier's ability to train themselves to go without sleep (*p* = 0.046), there were no significant differences in sleep knowledge/ attitudes between pre/ posttest.

**Table 2:** Sleep knowledge, attitudes, practices, beliefs, and perceived competence.

**Sleep knowledge**	**Pre-training**	**Post-training**	***Z***	***p***
It is normal for Soldiers to wake up a few times in the middle of sleeping	65%	74%	−2.731	0.006^*^
If Soldiers sleep more ahead of time, they can manage long work hours better	65%	74%	−1.884	0.059
Napping can interfere with sleep patterns	61%	71%	−1.178	0.239
If Soldiers don't get enough sleep during the week, they can try to recover by sleeping more on the weekends	32%	35%	−1.109	0.267
Soldiers should aim to get at least 7 h of sleep per day	97%	100%	−0.535	0.593
Soldiers should start to limit exposure to bright screens at least 1 h before lights out	94%	100%	−1.155	0.248
Alcohol can interfere with getting a good night's sleep	100%	100%	−1	0.317
Driving while drowsy can be similar to driving with an elevated blood alcohol level	97%	100%	−0.707	0.48
**Sleep attitudes**	**Pre-training**	**Post-training**	***Z***	***p***
Soldiers can train themselves to go without sleep	13%	6%	−1.999	0.046^*^
Sleep is primarily an individual responsibility	58%	61%	−0.077	0.939
Sleep deprivation is part of being a Soldier	23%	32%	−0.035	0.972
Healthy sleep in a unit is a responsibility shared by Leaders and Soldiers	100%	100%	0	1
Soldiers should be trained to manage their own sleep better	97%	97%	−0.905	0.366
Soldiers should be empowered to take responsibility for their sleep habits	100%	97%	−1	0.317
Soldiers should be aware of how much sleep they get	97%	100%	−1.89	0.059
**Sleep practices**	**Mean pre**	**Mean post**	***Z***	***p***
Sleep duration	6.831	7.06	−1.644	0.1
Sleep debt	0.677	0.724	−0.377	0.706
Sleep quality	0.555	0.561	−0.211	0.833
Effect of sleepiness/ tiredness	0.641	0.604	−1.12	0.263
**Sleep beliefs**	**Mean pre**	**Mean post**	***Z***	***p***
Sleep health	0.899	0.921	−1.806	0.071
**Perceived competence**	**Pre-training**	**Post-training**	***Z***	***p***
I feel competent in my knowledge pertaining to sleep	65%	87%	−2.519	0.012^*^
I am capable of educating Army leaders about sleep	68%	90%	−3.146	0.002^*^
I am able to incorporate sleep mgmt. techniques	74%	94%	−2.487	0.002^*^
I feel able to meet the challenge of managing sleep in my brigade	45%	61%	−1.625	0.013^*^

^*^p < 0.05.

### 3.3 Sleep practices and sleep beliefs

Comparisons in sleep practices and sleep beliefs are provided in Table 2. There were no significant differences between sleep duration subscale scores at baseline (6.83) and sleep duration posttest subscale scores (7.06-) (*p* = 0.100). Before receiving SLT, 42% of HPT members endorsed getting < 7 h of sleep per night on worknights (average 6.63 h) with 13% getting < 7 h of sleep on weekends/ holidays (average 7.3 h). Six weeks after the delivery of SLT, these numbers decreased to 39% (average 6.83 hours), and increased to 16% (average 7.60 h), respectively.

There were no significant differences in sleep debt subscale scores at baseline (0.677) compared to posttest scores (0.724) (*p* = 0.706). Accumulated sleep debt ranged from −0.14 (14 min more than needed per night) to 3.71 h pre SLT and −0.64 to 2 h post SLT. All other aspects of sleep practices including sleep quality as well as the effect of sleepiness/ tiredness were insignificant (*p* = 0.833 and *p* = 0.263 respectively). Finally, comparisons between subscale scores for sleep beliefs were also found to be insignificant (*p* = 0.071).

### 3.4 Perceived competence

Regarding perceived competence, scores for all four questions improved from pre to posttest, with each one demonstrating a significant change (Table 2). At the conclusion of the study, 39% of HPT members continued to feel unable to meet the challenge of managing sleep in their brigades (*p* = 0.013).

### 3.5 Participant interviews

Thirteen participants were interviewed by the first author and three co-authors aided with reviewing and refining 412 data extracts and 14 collated codes. The research team met virtually throughout the process to collectively refine analysis of the data. Four themes emerged from the data: (1) intrinsic motivation leading to self-directed learning, (2) military culture, (3) revenge sleep procrastination (RSP) and (4) structure and confidence (Table 3).

**Table 3:** Qualitative results from semi-structured interviews.

**Theme**	**Quote**
Intrinsic motivation leading to self-directed learning	“*…a topic that was probably touched on. Yeah. It was never really like, covered in depth, as far as a block of instruction or anything” (P5)*
	“*I did not have a lot of information as far as like from schooling, don't laugh but everything I learned about sleep was from that Joe Rogan podcast with that sleep scientist that was 2 h long” (P12)*
	“*…not a whole lot of specific education or focusing specifically on sleep, although it was always something that I would bring up. So, I started doing some research looking into the research, trying to read up on various studies, listening to like podcasts, different things like that” (P7)*
Military culture	“*…and then also mixed with the culture… you know, bragging about, I only got 4 h of sleep, oh yeah, well I got 3 h of sleep, you know, I'm fine. It's like a ton of the culture from what I've seen in the military… where you fit in more if you say you don't sleep, vs. saying I get a full night's sleep every night” (P8)*
	“*This organization feels that there should be a certain amount of quote, unquote, time that people are at work, and the idea that, when work is done to go home…. if it's before that quote unquote, no later than time, is, foreign” (P10)*
	“*I get the importance of PT and I think it's amazing, you know, looking at the population in America and how out of shape we are that people are doing the exercise, but now that's basically two and a half to three more hours on their day. By the time they get there, do their pt, do their hygiene, get back to work at nine, now they must work nine to five.” (P10)*
Revenge sleep procrastination	“*…the whole day they go without making any decisions on their own. And not getting to do any kind of fun or relaxation things… they probably turn to those video games to turn their brain off before they go to bed, or some of them use it to connect with their friends from back home…. So it's very hard to tell them like, Hey no, this is how you X YZ affects your sleep when you have no control over your environment, no control over your timeframe” (P9)*
	“*…usually I get 7 h at least, but when I don't, it can be because of that revenge, bedtime, procrastination…. wanting to do stuff on my own before I go to bed because when I get home, I don't have time for myself still until toward the end when my daughter's in bed” (P10)*
	“*…they're here for 12 h or something like that and then most of them have families so they then have to go home and be a dad or be a mom and then they have to try and sleep as well. So at that point in time, priority is, is not sleep. Priority is being a family member priority may be physical readiness to going out and exercising, priority may be just like, Hey, I need something just like downtime to just read or play a video game or something as opposed to sleeping” (P13)*
Structure and confidence	“*…having all the information condensed into one place to really be able to show leadership the impact that they have on the sleep for soldiers and the importance that, that carries, rather than having to piece together 20 or 30 different sources and filter through and figure it all out.”*
	“*I think that a structured way to educate others. Formatted structure gives leaders some confidence and say, Hey, this is the plan. This is how it's been executed from A to Z…. I feel empowered and confident and, in my ability, to speak intelligently on the matter”*
	“*…now when sleep questions come up or we're training, or if someone's out and I am assigned to teach sleep stuff, I feel really confident”*

#### 3.5.1 Intrinsic motivation leading to self-directed learning

Participants reported being underprepared to shift from their typical roles to that of a provider who addresses sleep. Many reported that their degree-granting programs did not include content related to the importance of sleep, or how to address sleep problems. HPT members expressed a desire to do well in their new roles, despite a lack of foundational education and many reported seeking out alternative methods to enhance their own knowledge about sleep. Some HPT members sought out other professionals to serve as peer-mentors or conducted personal research on the matter. Several HPT members reported reading books and listening to popular podcasts about sleep to enhance their skills in this area.

#### 3.5.2 Military culture

HPT members expressed challenges to addressing sleep in the Army due to the culture. They openly discussed unspoken rules such as feeling the need to stay late in the evening if one's supervisor was still present or staying late despite having no further work to do. They reported that SMs have routinely extended work hours that are accepted and normalized as part of their service to the nation, and that it was more common for SMs to get < 7 h of sleep a night, than >7 h. HPT members reported that work schedules are generally inefficient with many unproductive gaps in the day which increase the hours of work exponentially and leave SMs with little personal time to manage their own affairs.

#### 3.5.3 Revenge sleep procrastination

Several HPT members discussed the concept of *RSP* as a barrier to sleep. RSP, also referred to as *revenge bedtime procrastination* is defined by Kroese et al. (2016, p. 93) as “the phenomenon of postponing going to bed, typically resulting in a lack of sleep,” or an act of defiance against the increasing demands at home and at work, that leaves one with minimal time for leisure activities. HPT members reported recognizing SMs engaging in RSP response to having little personal control over their own day-to-day schedules. They noted that when SMs finally had the opportunity to sleep in the evenings, they often willingly delayed sleep, instead staying awake to participate in other activities such as playing video games, spending time with their spouses or children, or communicating with family members in different time zones. They discussed that SMs work long hours in stressful jobs and require time before bed to quiet their minds, in preparation for sleep. Serval of the HPT members interviewed endorsed RSP as a barrier to sleep in their own lives.

#### 3.5.4 Structure and confidence

Finally, HPT members reported benefiting from the structure and organization of SLT. They noted that the information they received from the training validated the information they had garnered through self-directed learning and that this affirmed the ways they had taught themselves to address sleep in their units, contributing to enhanced confidence. The packaged, condensed materials made it easy to present the topic of sleep and contributed to their comfort in teaching the materials despite their lack of foundational education on the topic.

## 4 Discussion

This study sought to determine the effect of SLT on the sleep-related knowledge, practices, attitudes, beliefs, and perceived competence of HPT members and to explore their experiences addressing SM sleep. Overall, the training had no significant effects on HPT members' sleep practices or sleep beliefs and limited effects on their attitudes and knowledge about sleep. Despite this, the training did appear to improve the perceived competence of HPT members to address the complex sleep issues faced by SMs.

HPT members in this study were selected for their positions despite 74% having no formal education related to sleep or sleep interventions. Much of the current evidence suggests that healthcare providers receive minimal education related to sleep and are inadequately prepared by their educational programs to address sleep (Ye and Smith, 2015; Meaklim et al., 2020; Abdelwadoud et al., 2022). Some authors suggest that this lack of preparedness might even be contributing to the sleep crisis that is occurring in the United States (Meaklim et al., 2020). Interviews with HPT members illuminated that they recognize their lack of preparedness and frequently sought out alternative ways to enhance their capabilities.

Regardless of their lack of formal education, when compared to general SMs, HPT members had higher sleep-related knowledge and better sleep-related attitudes at baseline. For example, Adler et al. (2021) assessed the knowledge of Army leaders prior to the delivery of SLT and found that only 22.9% agreed that it was normal for Soldiers to wake a few times in the middle of the night, whereas this number was as high as 65% in baseline measures of HPT members. When she assessed their attitudes, 28.6% of her leaders agreed that Soldiers can train themselves to go without sleep, whereas only 13% of HPT members agreed with this statement. Because HPT members were more educated about sleep at baseline, they may have had less room for improvement in their sleep-related knowledge and attitudes.

At baseline, HPT members were achieving a mean sleep duration approximately one-half hour less than the amount of sleep recommended by the American Academy of Sleep Medicine (2015). SLT had no significant effect on sleep duration in this study. Even after delivery of a sleep-educational intervention, almost 40% of HPT members, who are themselves, designated to address sleep in the Army, continued to be unable to achieve adequate sleep. HPT members interviewed in this study endorsed complex barriers to sleep in their own lives including long work hours, balancing work and family demands and difficulty detaching from work. The unique barriers to sleep faced by SMs are profound and will likely require much more than an educational course to overcome. This is congruenrt with current literature which demonstrated that that healthcare workers in general are known to be susceptible to insufficient sleep and disorders of their sleep (Hittle et al., 2023).

The concept of SMs engaging in RSP was an interesting finding in this study. Regularly extended hours at work may interfere with one's ability to perform needed personal activities *and* still achieve adequate sleep. It can be expected then that SMs who are required to routinely work beyond 8 h a day may need to delay their bedtime, thereby decreasing their sleep, to create the opportunity to engage in activities which provide one's life with purpose and meaning. HPT members endorsed that RSP occurs in both the SMs they treat, and in their own lives Sleep procrastination is common in non-military connected populations also. Kroese et al. (2016) found that over 53% of young adults reported delaying sleep to engage in watching television, using computers, or socializing.

At baseline, more than half of HPT members felt competent in their sleep-related knowledge, their ability to educate leaders about sleep, and their ability to incorporate sleep management practices, however, only 45% felt capable of managing sleep issues in their brigade. SLT had a significant effect on these perceived competencies. Competence is enhanced by education or experience which provides advanced knowledge, traits skills and/or abilities (Kak et al., 2001). HPT members appeared to benefit from the opportunity to receive, what was for many, their first formal sleep education, and the opportunity to practice the delivery of sleep education to Army leaders in this virtual group setting. In their interviews, HPT members regarded the structure and format of the training, as well as the provided materials as empowering, and noted it contributed to their competence in their ability to address sleep with Army leaders.

### 4.1 Study limitations and recommended future research

One of the main limitations of this study is that it lacked the use of a control group. It was not possible to utilize a control group in this study as the training was mandated for *all* HPT members. Future studies may wish to consider randomized controlled trials which compare the effects of SLT to other sleep education programs. Further, we used only self-report questionnaires. Self-reported data can be subject to several types of biases. Future studies may consider using actigraphy or wearable sleep devices to assess any objective differences in measures of sleep health after SLT.

Strengths of this study include a high retention rate and collection of follow-up data a month after training. Unfortunately, no outcome measures exist which adequately capture nuances specific to military populations, such as the impact of a palpable but immeasurable culture. In fact, many commonly used psychosocial, or sleep outcome measures are not validated for use in military populations. This may be a goal for researchers interested in understanding sleep in military SMs. Our use of a mixed-methods design added depth and context to questionnaire results. The qualitative findings from this study can be particularly helpful for Army leaders, healthcare workers and policy makers. This study utilized a brief (two day), multimodal (mix of didactic and practicum) virtual (computer-based) means of delivering sleep education. Virtual education has expanded significantly, although its utility and efficacy remain questionable. This study demonstrated that a virtual intervention resulted in significant improvements in perceived competence. Because sleep education is sparse in many degree-granting programs, delivering this education virtually may be an effective means of enhancing the number of healthcare providers who are both qualified and competent to address sleep in this unique population.

## 5 Conclusion

To the authors' knowledge, this is the first study to assess the effect of SLT on the sleep characteristics of Army HPT members. The study demonstrated that SLT is an effective method of enhancing the perceived competence of Army HPT members to address sleep, despite a lack of foundational training. Most SMs and HPT members get less than the recommended amount of sleep per night. HPT members endorsed the same barriers to sleep faced by the SMs they treat. The military imposes unique challenges to sleep, even for those responsible for managing the sleep readiness of the force. Additional research is needed to determine what interventions might be most effective for overcoming longstanding cultural barriers to sleep in this unique subset of the population.

## Data availability statement

The datasets presented in this article are not readily available because data is property of a U.S. Government employee. Requests to access the datasets should be directed to carlyrcooper@yahoo.com.

## Ethics statement

The studies involving humans were approved by San Antonio Review Board/Brooke Army Medical Center. The studies were conducted in accordance with the local legislation and institutional requirements. The participants provided their written informed consent to participate in this study.

## Author contributions

TB: Conceptualization, Data curation, Formal analysis, Investigation, Methodology, Resources, Software, Writing – original draft, Writing – review & editing. BY: Data curation, Investigation, Methodology, Software, Writing – review & editing. CC: Data curation, Formal analysis, Methodology, Software, Writing – review & editing. ED: Data curation, Formal analysis, Investigation, Supervision, Writing – review & editing. JU: Formal analysis, Methodology, Software, Writing – review & editing. BG: Investigation, Methodology, Resources, Writing – review & editing. MB: Writing – review & editing.
